# Virome analysis for identification of novel mammalian viruses in bats from Southeast China

**DOI:** 10.1038/s41598-017-11384-w

**Published:** 2017-09-07

**Authors:** Dan Hu, Changqiang Zhu, Yi Wang, Lele Ai, Lu Yang, Fuqiang Ye, Chenxi Ding, Jiafeng Chen, Biao He, Jin Zhu, Hui Qian, Wenrong Xu, Youjun Feng, Weilong Tan, Changjun Wang

**Affiliations:** 1 Department of Epidemiology, College of Preventive Medicine, Third Military Medical University, Chongqing, 400038 China; 2 Department of Epidemiology, Research Institute for Medicine of Nanjing Command, Nanjing, 210002 China; 3 Key Laboratory of Jilin Province for Zoonosis Prevention and Control, Institute of Military Veterinary, Academy of Military Medical Sciences, Changchun, Jilin, China; 4 Key Laboratory of Laboratory Medicine of Jiangsu Province, School of Medicine, Jiangsu University, Zhenjiang, 212013 Jiangsu China; 5 College of Animal Sciences, Zhejiang University, Hangzhou, 310058 Zhejiang, China

## Abstract

Bats have been shown as important mammal resevoirs to carry a variety of zoonotic pathogens. To analyze pathogenic species in bats from southeast coastal regions of China, we performed metagenomic sequencing technology for high throughput sequencing of six sentinels from southeast coastal area of China. We obtained 5,990,261 high quality reads from intestine and lung tissue of 235 bats, including 2,975,371 assembled sequences. 631,490 reads predicted overlapping sequences for the open reading frame (ORF), which accounts for 2.37% of all the sequences (15,012/631,490). Further, the acquired virus sequences were classified into 25 viral families, including 16 vertebrate viruses, four plant viruses and five insect viruses. All bat samples were screened by specific PCR and phylogenetic analysis. Using these techniques, we discovered many novel bat viruses and some bat viruses closely-related to known human/animal pathogens, including coronavirus, norovirus, adenovirus, bocavirus, astrovirus, and circovirus. In summary, this study extended our understanding of bats as the viral reservoirs. Additionally, it also provides a basis for furher studying the transmission of viruses from bats to humans.

## Introduction

Bats belong to the order Chiroptera, and are the second largest order of mammals after rodents. This order includes 19 families and 962 species distributed across the globe^1^. More than 130 kinds of viruses have been detected in bats, including more than 60 species of zoonotic viruses which are highly pathogenic in humans^2^, such as severe acute respiratory syndrome (SARS)-like coronavirus (SL-CoV), Ebola virus, Nipah virus, and Hendra virus^3–6^. In 2002, SARS outbreak in China infected more than 8,000 people worldwide and killed at least 800 people. Chinese horseshoe bats were proved to be the natural reservoirs of SARS-CoV and that intermediate hosts may not be necessary for direct human infection by a bat hosting SL-CoV^7^. Similarly, Middle East respiratory syndrome (MERS)-associated coronavirus were detected in bats, and required dipeptidyl peptidase 4 cell receptors for its invasion into host cells^8^. It seems likely that bats might act as natural hosts which critical roles in viral iner-host transmission.

In the last five years, the use of second-generation sequence technology allowed us to elucidate a flood of viruses, like SARS-CoV, hepatitis B virus, rotavirus and other important viruses^9–11^. In 2010, meta-genomic analysis was conducted for the first time, following the second generation sequencing of oral swab and faecal samples from 41 bats of three common North American bat species. The results showed that sample pools contained strong matches to at least three novel group of CoVs, and large numbers of insect and plant virus sequences were identified^12^. One bat virome analysis conducted by Ge *et al*. on fecal samples of bats from six locations in China^13^, and 97 contigs were found to be related to eukaryotic viruses, including coronavirus. Then, one bat virome analysis in Myanmar conducted by He *et al*. has identified many new mammalian viruses of Myanmar bats^14^, showing that the composition of bat viromes differs depending on geographical location and bat species. These studies show that the study of the bat viruses by the metagenomic analysis can be insightful.

The coastal wetlands southeast of China consist of growing ports, industrial districts, and port cities. Studies show the occurrence of natural focal diseases, such as Dengue fever and hemorrhagic fever with renal syndrome, in species-rich and densely populated southeast of China^15, 16^. It is important to understand the distribution of pathogens in different animals across different habitats. An understanding of the natural habitat of bat-associated viruses can prevent newly emerging and re-emerging zoonoses. To expand these studies to southest China, Here we combined second-generation sequencing technology with meta-genomics to understand the outbreak of new infectious diseases caused by animal-origin pathogens and explore the unknown viruses from the natural environment, humans, and animals. In this study, we collected 235 bats from six locations in the southeastern coastal area of China from July 2015 to August 2015. Using Illumina platform for sequencing gut and lung tissue from bats, we detected a total of 25 species of the virus family, including norovirus, which was detected first. Also, we sequenced *Myotis formosus* for the first time from the southeastern coastal area and found that astrovirus. This work extended our understanding the diversity of bats harboring virsues and provide new clues to monitor these transmittable zoonotic viruses.

## Results

### Sampling

**Figure 1:**
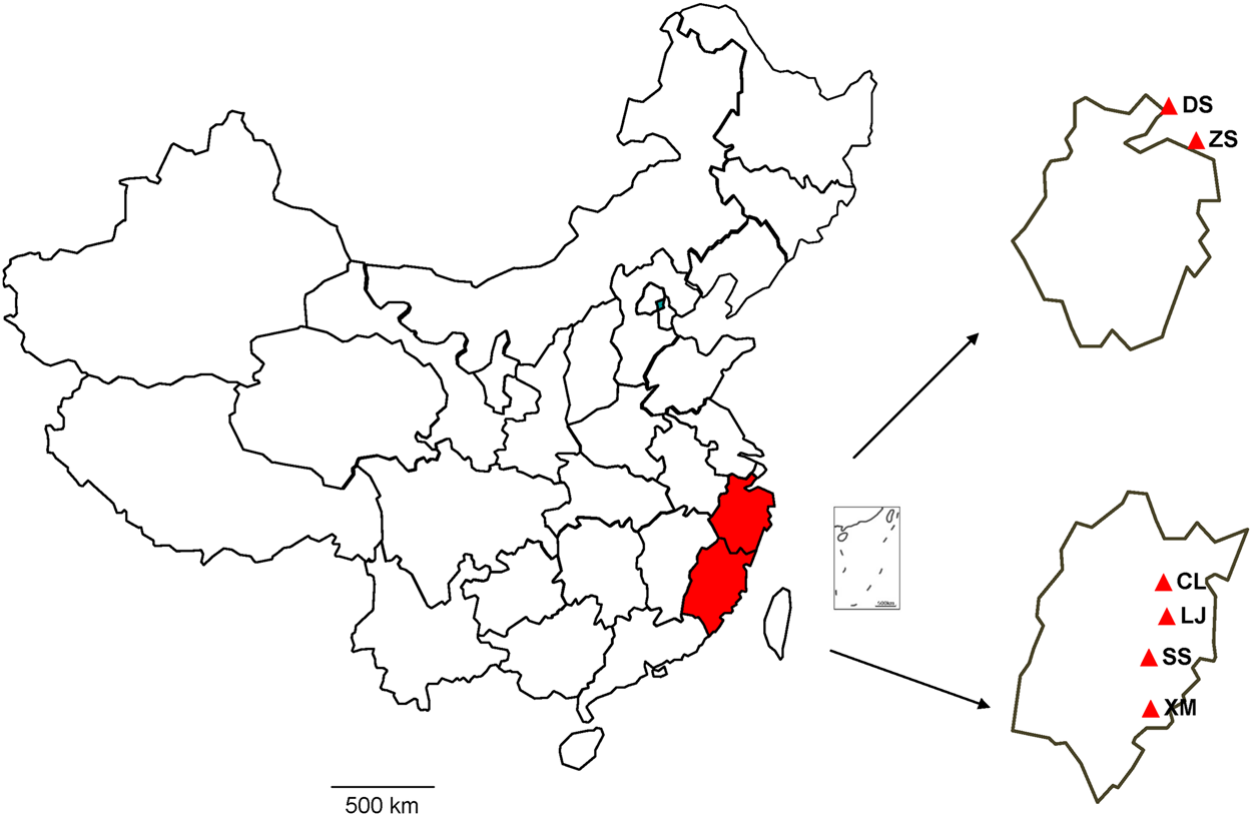
Overview of the sample collection. Map showing the location of bats collected in this study (labeled in red; ZS = Zhoushan, DS = Daishan, CL = Changle, LJ = Lianjiang, SS = Shishi, XM = Xiamen). The map of China was created and processed using the software of Adobe Illustrator CS6.

A total of 235 adult bats were captured live with nets near or in human dwellings from Zhoushan (ZS), Daishan (DS), Xiamen (XM), Changle (CL), Shishi (SS), and Lianjiang (LJ) between July to August in 2015 in Zhejiang and Fujian Provinces of China (Fig. 1). All bats looked healthy and had no obvious clinical signs at capture. Based on the mitochondrial cytochrome *b* gene, the bats can be divided into five species based on their muscle tissues: *Rhinolophus ferrumequinum, Myotis formosus, Scotophilus kuhlii, Myotis davidii*, and *Rhinolophus pusillus*. All intestine and lung samples were classified and combined into 12 pools and then subjected to metagenomic sequencing.

### High throughput sequencing

The original data was processed with in-house scripts. After quality control and host read removal, a total of 5,990,261 reads were employed for further analysis. The read length was between 80 bp and 100 bp. For each sample, an average of 89.85 Mbp data were obtained (range: 48.16Mbp-167.31Mbp). The percentage of bases with a quality score of > = Q20 relative to the total bases in each sample ranged from 94.23% to 96.70% (mean percentage: 95.35%). The corresponding percentages of high-quality bases (> = Q30 and > = Q40) in each sample ranged from 87.57% to 92.34% (mean percentage: 89.76%) and from 62.41% to 72.80% (mean percentage: 67.57%), respectively. The filtered clean data matched with the NCBI bacterial genome, fungal genome, and virus database. Reads on the reference database were compared, and 327,159 reads were detected as bacterial sequences, 298,008 reads (4.93%) were detected as eucaryon sequences, 51 reads (0.00085%) were detected as archaeal sequences, and 2,180 reads (0.036%) were detected as virus sequences. 49.67% (2,975,37/5,990,261) reads were successively assembled into 2,975,371 overlapping sequences with an average read length of 140–150 bp. A total of 631,490 tags were predicted as ORF, where the genes were aligned through the BLAST software and NCBI virus database. A total of 15,012 tags (2.3%) of functional annotations matched virus sequences (Table 1) after classification by MetaGenome Analyzer (MEGAN).

**Table 1:** Overview of Solexa sequencing.

Group	Total pairs read number	Archaea	Bacteria	Eukaryota	Viruses	Connect Read Number	ORFs	Virus ORFs
ZS	1266477	9	19051	74072	605	717963	137926	11692
DS	569424	6	26511	61441	110	357111	57555	112
XM	1058551	16	29455	74491	727	639948	158797	474
CL	1536852	9	248326	13856	541	601862	122082	2537
SS	668392	8	2932	60763	77	289163	49862	87
LJ	890565	3	884	11105	120	369324	105268	110
Total	5990261	51	327159	295728	2180	2975371	631490	15012

### Virus classification

**Figure 2:**
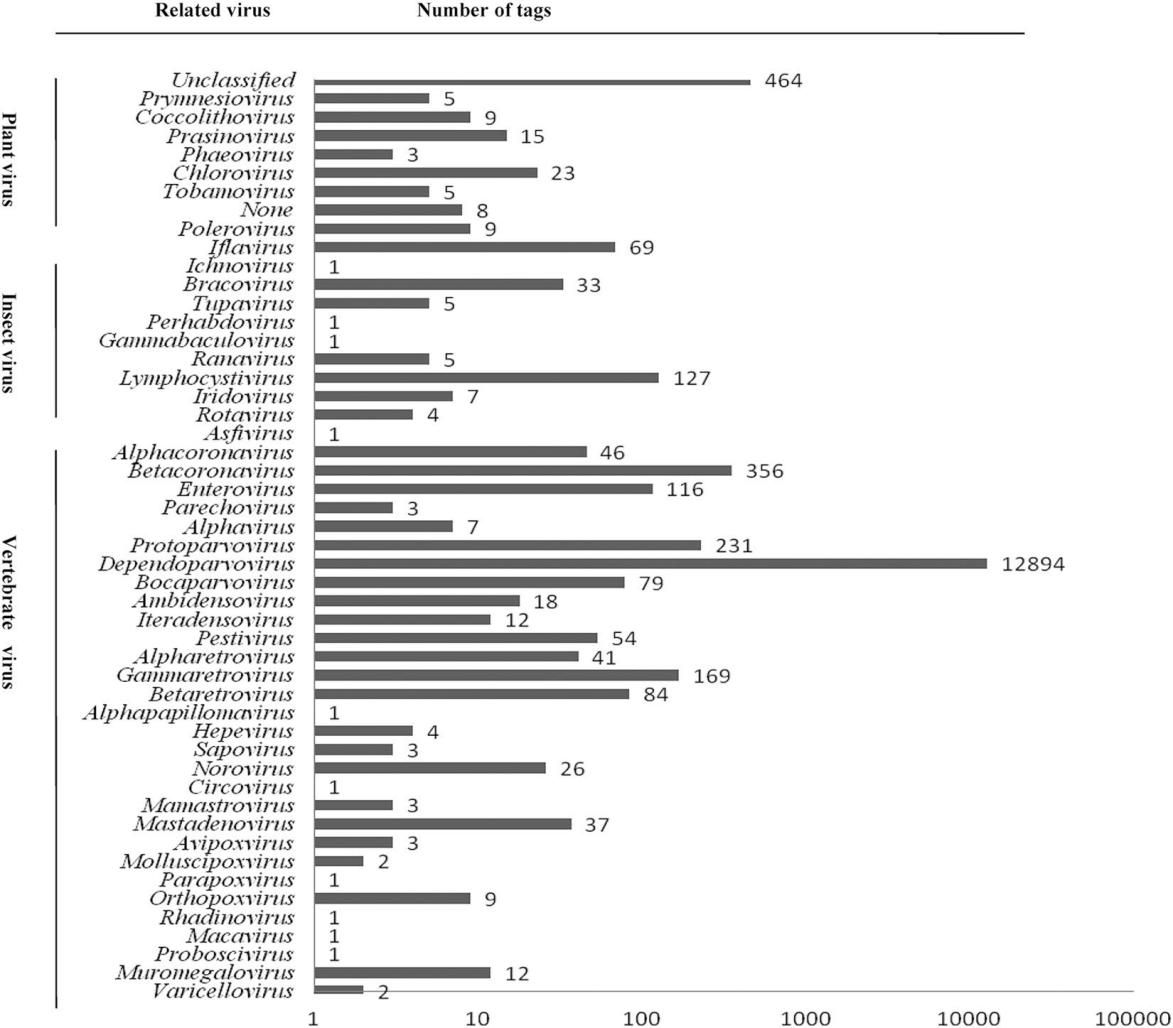
Overview of the viral tags in each pooled sample. The numbers of viral tags from each group are described in Supplementary Table S1.

The 15,012 viral tags were classified into 25 virus families (Fig. 2). About 94.7% (14222/15012) of tags were annotated for the mammalian families, including a total of 16 virus families: *Herpesviridae*, *Coronaviridae*, *Poxviridae*, *Picornaviridae, Adenoviridae, Asfarviridae, Astroviridae, Caliciviridae, Circoviridae, Hepeviridae, Papillomaviridae, Reoviridae, Retroviridae, Flaviviridae, Parvoviridae*,and *Togaviridae*. The proportion of viruses in this study was not the same compared with previous research^13, 14, 17^. The proportion of *Parvoviridae* accounted for a maximum of 88.16% (13234/15012) in vertebrate virus, which accounted for more than 97.43% (12894/13234) in the *Parvoviridae* was further annotated as *Dependoparvovirus*, followed by the 2.67% *Coronaviridae* (402/15012). The remaining *Herpesviridae, Poxviridae, Picornaviridae, Retroviridae*, and *Flaviviridae* existed in each group, indicating that viruses are a common presence in bats aspreviously reported. Although the viruses *Adenoviridae, Circoviridae, Asfarviridae*, and *Caliciviridae* annotated to a small number of sequences (<40 tags), but NCBI results show that known viruses exhibit low nucleotide (nt) or amino acid (aa) sequence identities.

Insect virus families contributed to 1.66% (249/15012) tags, including the following virus families: *Baculoviridae, Iridoviridae, Rhabdoviridae, Polydnaviridae*, and *Iflaviridae*. Among them, *Iridoviridae* accounted for the most, with 49.8% of insect virus sequences. Iridovirus was originally detected in insect body, after isolating from several other animals, especially aquatic animals. Also, Iflavirus tags had >78% nucleotide identity with the Perina nuda virus of genus *Iflavirus*.

Plant viruses accounted for 0.51% (77/15012) of annotated tags, which included the following four viral families: *Luteoviridae, Totiviridae, Virgaviridae*, and *Phycodnaviridae*. *Phycodnaviridae* accounts for the largest proportion of plant viruses (77%). *Phycodnaviridae* virus infection is widely present in fresh water algae or seaweed across the world.

It is worth mentioning that 35.27% virus sequences shared low similarity with known viral sequences. To verify the reliability of metagenomic sequencing results, six viruses (coronavirus, mastadenovirus, mamastrovirus, circovirus, norovirus, and bocavirus) closely related to human disease were selected, and nucleic acid from bat tissue samples of all 235 bats was amplified by PCR (Table 2). Among the five species of bats, *Rhinolophus pusillus* carried the broadest spectrum of viruses including coronavirus, adenovirus, norovirus, circovirus, and astrovirus. Of the remaining four bat species, bocavirus was detected in the bat species *Myotis davidii* for the first time (N = 2), coronavirus was detected in the bat species *Rhinolophus ferrumequinum* (N = 2), andastrovirus was detected in bat species *Myotis formosus* (N = 1). However other viruses were absent.

**Table 2:** Number of bats species tested and validation summary of selected viruses.

Virus	ZS	DS			XM		CL		SS	LJ
	*R. pusillus*	*M. formosus*	*R. pusillus*	*M. davidii*	*R. pusillus*	*S. kuhli*	*R. pusillus*	*R. ferrumequinum*	*R. pusillus*	*R. pusillus*
	N = 45	N = 25	N = 14	N = 10	N = 19	N = 8	N = 44	N = 11	N = 35	N = 24
Coronavirus	7	0	0	0	0	0	3	2	4	0
Mastadenovirus	0	0	0	0	0	0	3	0	0	0
Bocavirus	0	0	0	2	0	0	0	0	0	0
Norovirus	9	0	0	0	0	0	1	0	0	0
Circovirus	1	0	0	0	0	0	1	0	0	1
Mamastrovirus	1	1	0	0	0	0	2	0	0	0

### Detection and identification of Coronavirus

**Figure 3:**
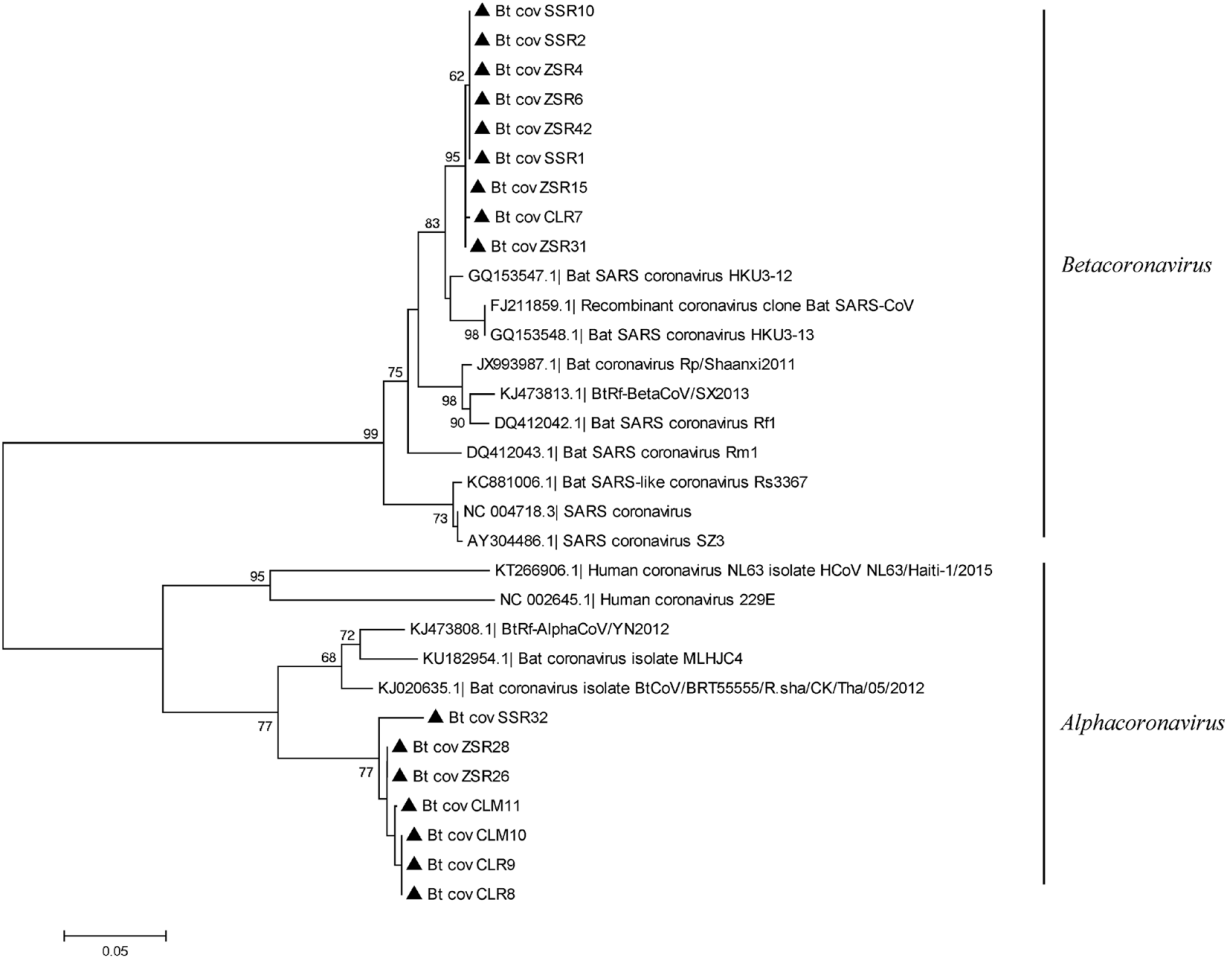
Phylogenetic analysis of 440 bp long region of the RNA-dependent RNA polymerase gene of bat coronavirus with other representatives, including alphacoronavirus and betacoronavirus. The tree was generated by using the neighbor-joining method with the Kimura 2-parameter model. A bootstrap test was replicated 1000 times. Numbers above the branches indicate NJ bootstrap values. Bold triangles indicate bcavirus detected in this study.

Coronaviruses are positive-sense,single stranded RNA viruses with the largest RNA genome, they belong to *Coronaviridae* family and *Coronavirinae* subfamily, and are divided into *Alphacoronavirus*, *Betacoronavirus, Gammacoronavirus*, and *Deltacoronavirus*. *Alphacoronavirus* and *Betacoronavirus* are present in mammals, while *Gammacoronavirus* and *Deltacoronavirus* are mainly present in livestock and poultry^18^. We found 62 *Alphacoronavirus* tags and 355 *Betacoronavirus* tags distributed in ZS, CL, and SS groups (Supplementary Table S1). All bat samples were amplified by a nested PCR which targeted a 440 bp fragment of conserved protein RdRp; 16 positive amplifications were detected from ZS, CL, and SS groups. The ZS group had seven out of 45, CL group had five out of 55, SS group had four out of 35 positive amplifications from *Rhinolophidae* family, including 14 cases of *Rhinolophus pusillus* and two cases of *Rhinolophus ferrumequinum*. Subsequently,16 positive amplifications were further sequenced and phylogenetically analyzed against *Alphacoronavirus* and *Betacoronavirus* samples (Fig. 3). Nine sequences shared 91.4%–99.8% identity to *Betacoronavirus*, SARS coronavirus SZ3, and SARS-related bat coronavirus in a large branch, while the remaining seven sequences belonged to the *Alphacoronavirus*, along with BtRf-AlphaCoV/YN2012, and Human coronavirus 229E in a large branch.

### Detection and identification of Adenovirus

**Figure 4:**
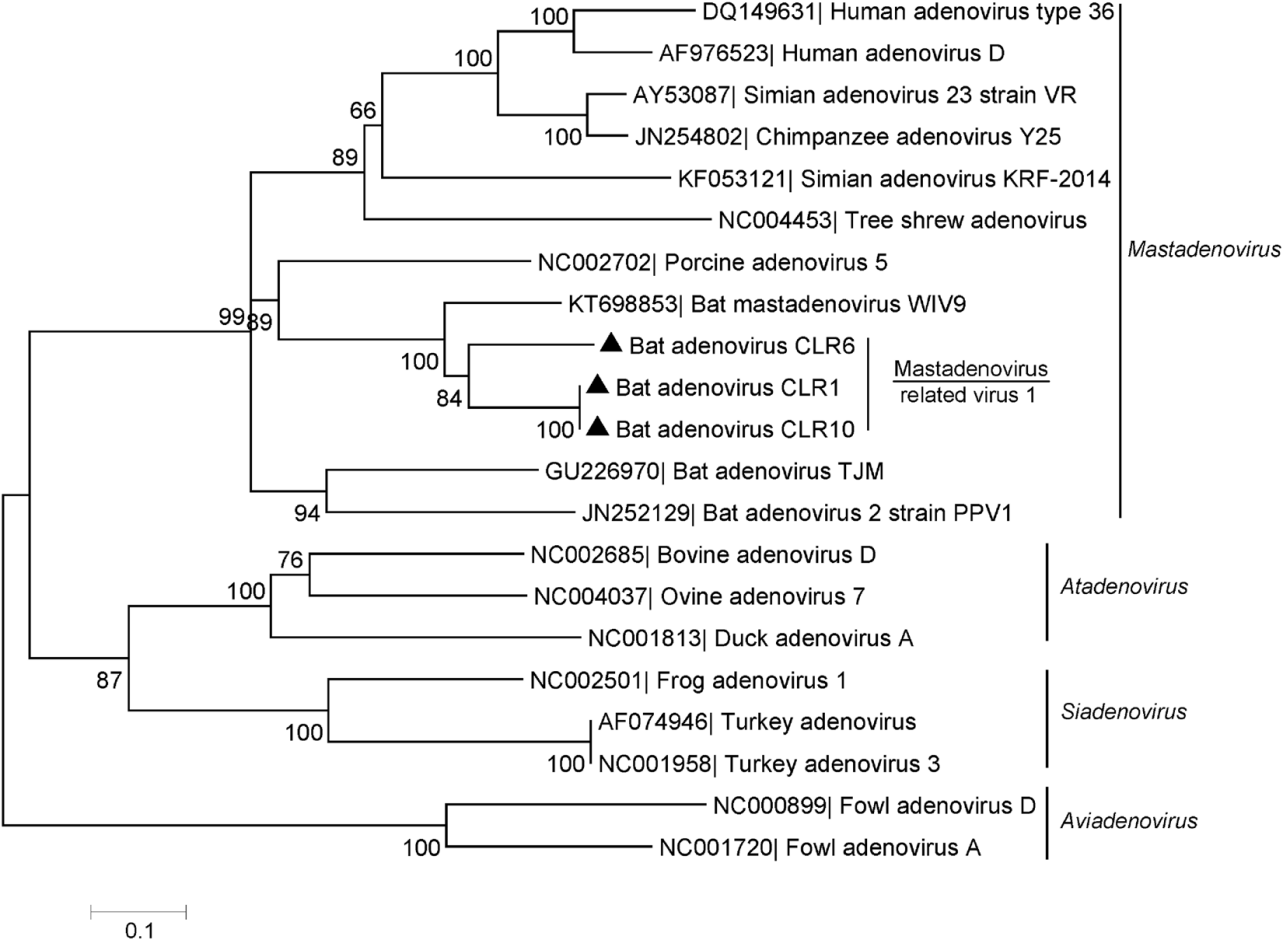
Phylogenetic analysis of bat adenovirus CLR1/6/10 and other representatives based on a 766 nucleotide segment of hexon. The tree was generated using the neighbor-joining method with the Kimura 2-parameter model. A bootstrap test was replicated 1000 times. Numbers above the branches indicate NJ bootstrap values. Bold triangles indicate adenoviruses detected in this study.

Adenoviruses (AdVs) are one of the most common viruses that infect humans and animals. These infections cause a variety of sub-clinical to lethal symptoms and outcomes^19^. In this viral metagenomic analysis, we found 21 tags showing identity with genus *Mastadenovirus* in group ZS, one tag showing identity with genus *Mastadenovirus* in XM group and 15 tags showing identity with genus *Mastadenoviru*s in CL group (Supplementary Table S1). Further screening of intestine from the bat samples by performing PCR of a partial hexon gene sequence of mammalian adenovirus (766 nt) confirmed that three sequences existed in the intestine of *Rhinolophus pusillus* from the CL group but not the other two groups. At present, partial genomic sequences(10603 bp) of bat adenovirus CLR6 were obtained by using degenerated primers, including three major ORFs (E1B1, Iva2,Pol, Genbank:MF278269). Phylogenetic analysis based on a partial hexon gene sequence (Fig. 4) showed that bat adenoviruses, CLR1, CLR6, and CLR10 shared 80.0% to 98.2% nucleotide sequence identity to each other and 55.1% to 78.7% nucleotide sequence identity with bat adenovirus as previously reported in China and Germany^20–22^. Therefore, the three bat adenoviruses were termed as Mastadenovirus-related virus 1.

### Detection and identification of Astroviruses

**Figure 5:**
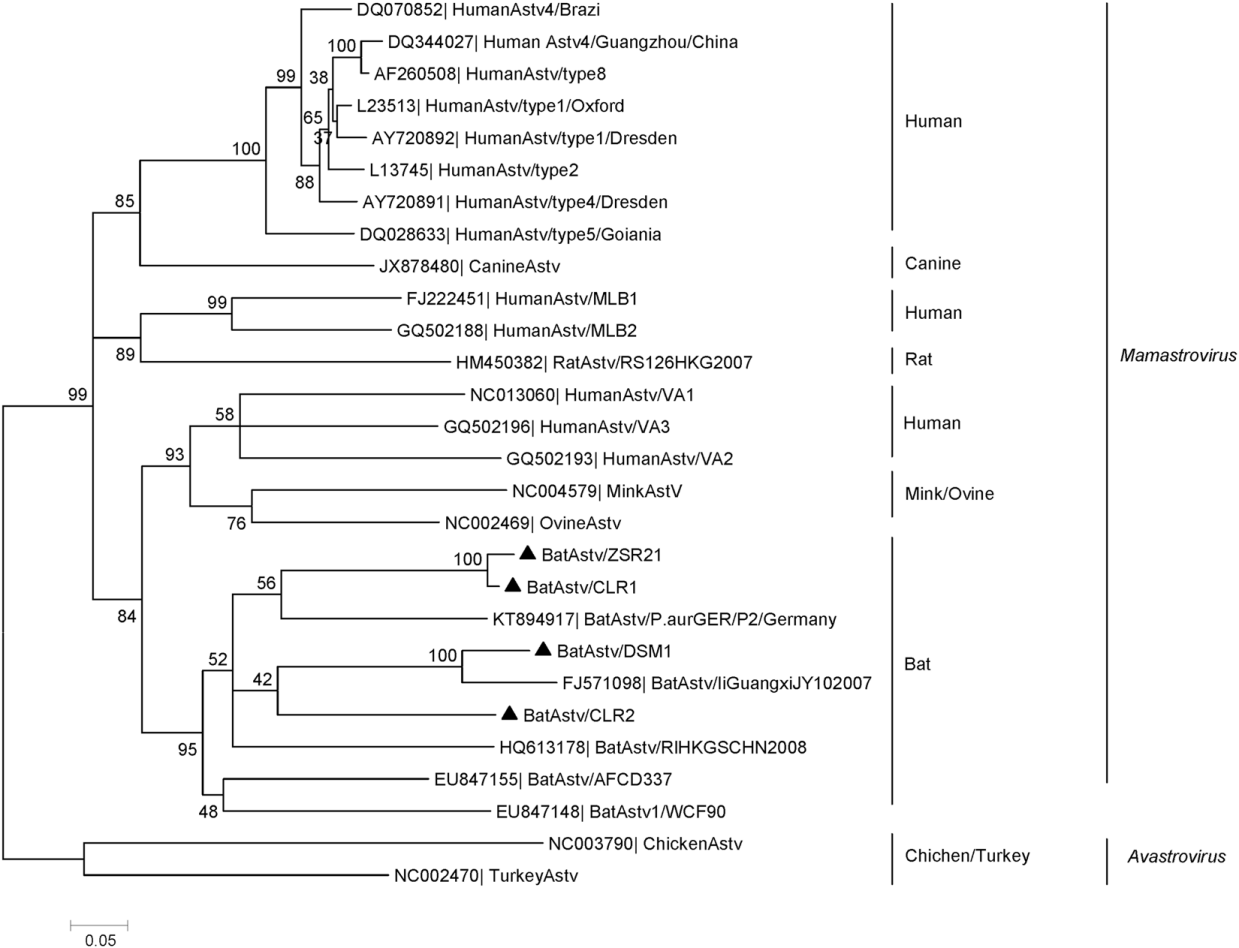
Phylogenetic analyses of novel astroviruses (BatAstv) identified from bats. The phylogenetic tree was constructed based on a 403 bp long region of the RNA-dependent RNA polymerase gene. The tree was generated by using the neighbor-joining method with the Kimura 2-parameter model. A bootstrap test was replicated 1000 times. Numbers above the branches indicate NJ bootstrap values. Bold triangles indicate adenoviruses detected in this study.

Mamastrovirus in the family Astroviridae infects many mammals, including humans, and causes gastroenteritis. In our study, three tags related to the genus *Mamastrovirus* were obtained from CL group (Supplementary Table S1). We tested all the bat intestine by hemi-nested PCR using pan-bat astrovirus primers which targeted a 422 nt fragment of ORF1b^23^. We found that ZS, DS, and CL groups had one, one, and two positive amplifications in the intestine sample. The positive amplification rate in *Rhinolophus pusillus* was 1.6% (3/187) in ZS and CL groups. In group DS, *Myotis formosus* showed positive amplification in 4% (1/25) of samples, while all other bat species were negative. We sequenced four amplicons and identified four bat AstVs. These four bat AstVs showed 65.4% to 96.7% nucleotide sequence identity to each other and 61.6% to 87.1% nucleotide sequence identity with bat AstVs as reported previously in Hong Kong, Guangxi, and Germany (Fig. 5).

### Characterization of Circovirus

**Figure 6:**
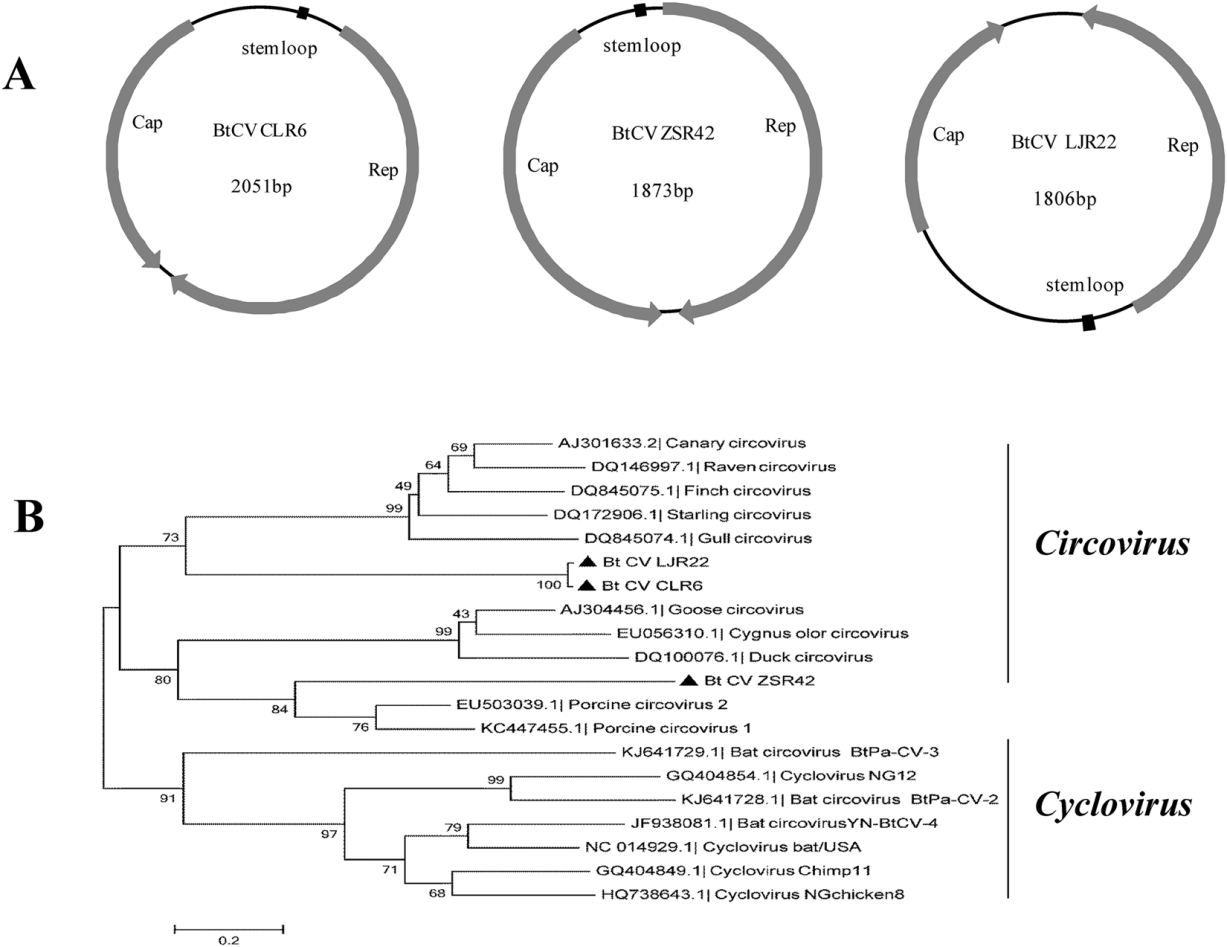
(**A**) Genome structures of BtCV-CLR6, BtCV-ZSR42, and BtCV-LJ22 (**B**). Phylogenetic analysis of complete sequence of circovirus and other representatives of circoviruses. The phylogenetic tree was constructed based on the complete sequence. The tree was generated by using the neighbor-joining method with the Kimura 2-parameter model. A bootstrap test was replicated 1000 times. Numbers above the branches indicate NJ bootstrap values. Bold triangles indicate circovirus detected in this study.

Members of the family *Circoviridae* are non-enveloped icosahedral particles with a diameter of 18-25 nm and a circular single-stranded DNA, approximately 2 kb in size^24^. In the present study, only group CL contained circovirus (CV) tags (Supplementary Table S1). Further screening of all the bat samples by PCR of a partial *Rep* gene sequence (550 bp) of circovirus confirmed that this sequence existed in the guts of *Rhinolophus pusillus* of groups ZS, CL, and LJ, but not the other groups. Three complete genomes BtCV ZSR42, BtCV LJR22and BtCV CLR6 (accession number: MF276911, MF278661, and MF322629) were amplified by inverse PCR. Sequencing revealed that BtCV ZS62, BtCV LJ22 and BtCV CL6 DNAs were respectively 1,873 nt, 1,806 nt, and 2,051 nt in length and contained two ORFs in opposite strands (Fig. 6A). Phylogenetic analysis was based on complete sequence (Fig. 6B) and multiple alignments showed Bt CV ZSR42 shared47.1% and 51.8% identity with Bt CV CLR6 and Bt CV LJR22, indicating that they were different isolates. Bt CV ZSR42 shared the highest circovirus identities to Porcine circovirus 2 (PCV 2)(37%) and Porcine circovirus 1(PCV1)(36.9%). Thus, Bt CV CLR6, BtCV LJR22 and Finch circovirus should be clustered into the genus *Circovirus*.

### Detection and identification of Bocavirus

**Figure 7:**
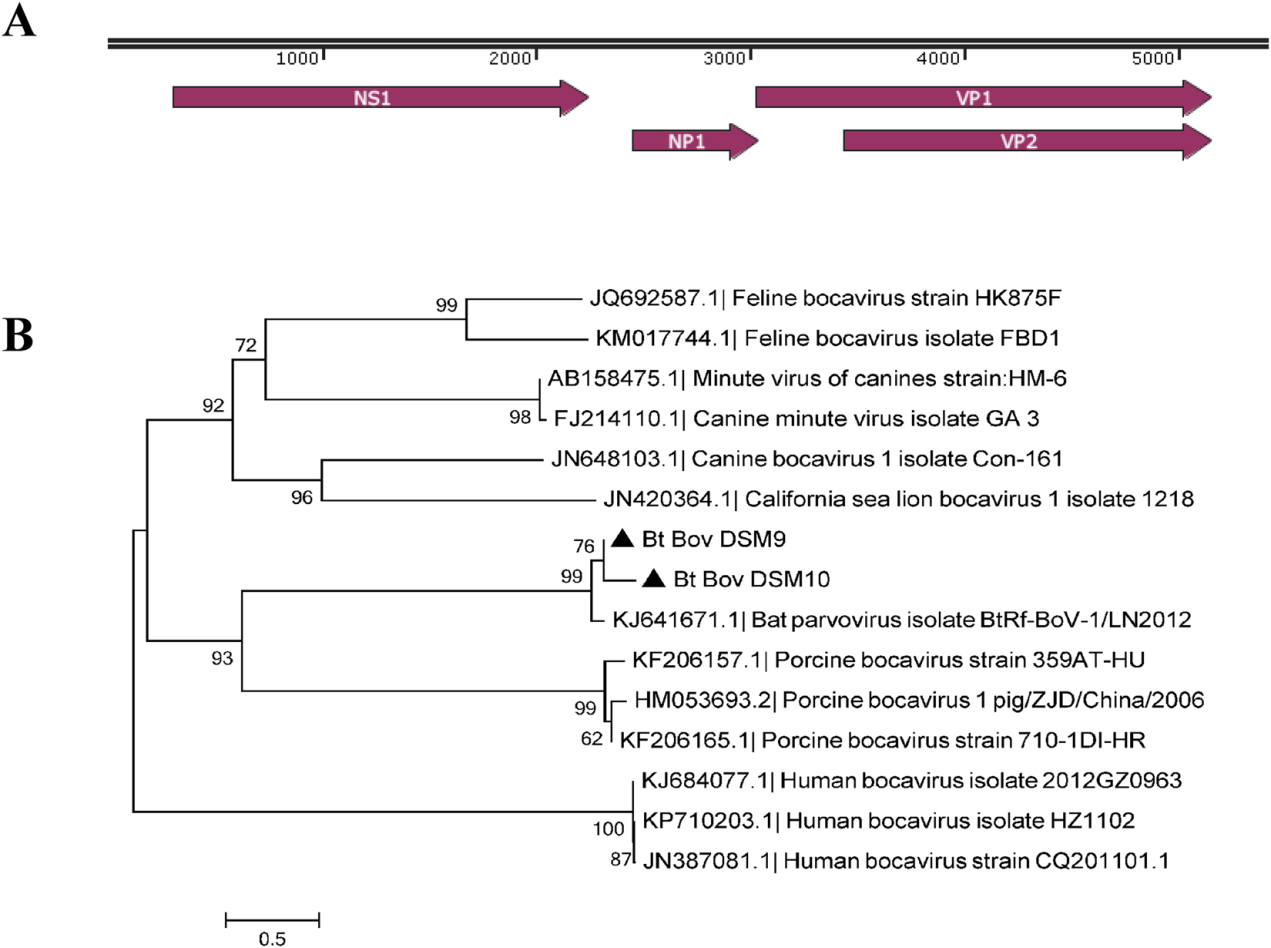
(**A)** Genome structures of bcavirus **(B**). Phylogenetic analysis of bat bocaviruses and other representatives. The phylogenetic tree was constructed based on partial *VP1* gene sequences deduced from 620 bp amplicons of bat bocaviruses. The tree was generated by using the neighbor-joining method with the Kimura 2-parameter model. A bootstrap test was replicated 1000 times. Numbers above the branches indicate NJ bootstrap values. Bold triangles indicate bcavirus detected in this study.

Bocavirus was first discovered in animals in the early 1960s, and subsequently it was found in cows, dogs, cats, and gorilla^25–27^. In this study, a total 45 tags were related to genus *Bocavirus*. Further screening of all the bat samples by performing PCR of gene sequence(550 bp) of partial capsid proteins(VP) in bocavirus confirmed the existence of this sequence in guts of *Myotis myotis* of the group DS but none of the other groups. The two positive samples were named Bt BOV DSM9 and Bt BOV DSM10. Nearly whole sequence of Bt BOV DSM10 (about 3,921nt, accession number: MF278662) were amplified by Genome Walking including three major ORFs (partial *NS*, *VP*, and *NP* gene). Phylogenetic analysis based on partial VP nucleotides and multiple alignments (Fig. 7) showed that the Bt BOV DSM10 shared 86% identity with Bt BOV DSM9, and Bt BOV DSM10 shared 74% identity with BtRf-Bov. The homology of Bt BOV DSM9 and Bt BOV DSM10 to Human bocavirus ranged from 46% to 47%, and the homology to Porcine bocavirus was between 58% and 62%. According to The International Committee on Taxonomy of Viruses (ICTV) criterion, bocavirus with 85% nt identity in its NS gene can be defined as a new species, and Bt BOV DSR9 and Bt BOV DSR10 can form a new species within the genus *Bocavirus*.

### Detection and identification of Norovirus

**Figure 8:**
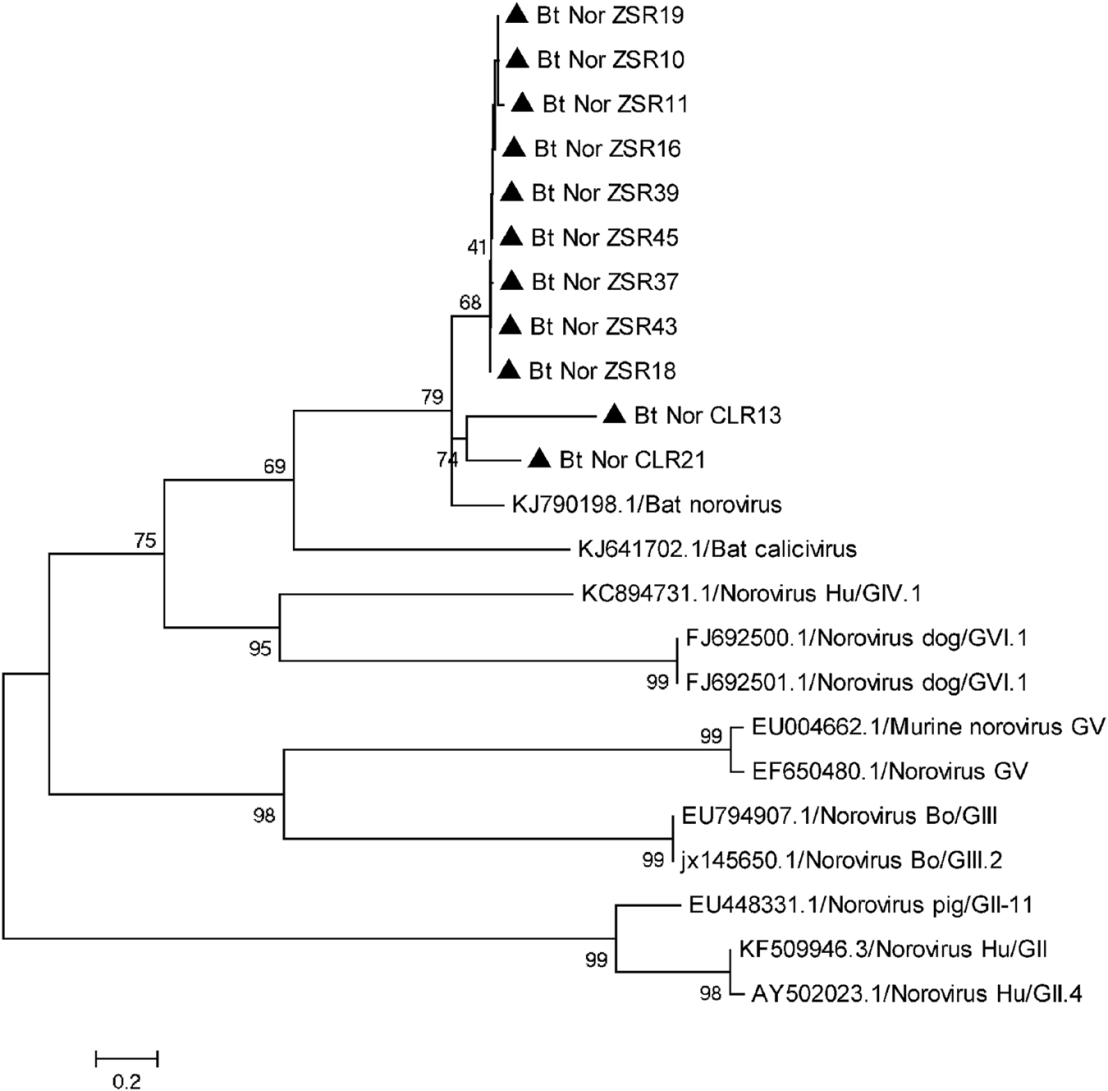
Phylogenetic analysis of partial *RdRp* gene sequences deduced from 660 bp amplicons of bat norovirus and other representatives. The tree was generated by using the neighbor-joining method with the Kimura 2-parameter model. A bootstrap test was replicated 1000 times. Numbers above the branches indicate NJ bootstrap values. Bold triangles indicate bcavirus detected in this study.

Norovirus (NV) is a representative strain of the genus *Norovirus* of family Human Calicivirus (HuCV), which is an icosahedron symmetric single-stranded positive-sense RNA virus with a diameter about 26 to 35 nm^28^. According to the sequencing results, degenerate primers were designed based on the conserved protein with 660 bp of norovirus RdRp. All the samples were screened by PCR resulting in nine positive amplifications of group ZS and two positive amplifications of group CL in the intestine. *Rhinolophus pusillus* showed positive amplification in 6.21% (11/177) of the sample, while all the other bat species were negative. 11 amplicons were further sequenced and phylogenetically analyzed against representative noroviruses of the four genotypes based on their nucleotide sequences (Fig. 8). However, bat norovirus detected in this study could be further grouped into two branches with 66.3%-78.4% nt identity between them. Nine noroviruses amplified from ZS shared the highest identity (99%) with each other. The positive viruses amplified from *Rhinolophus pusillus* shared the highest identity (84%) with bat norovirus from YunNan of China,and they were likely a new norovirus species based on the new ICTV criteria.

## Discussion

In this study, we collected samples for metagenomic analysis from southeast coastal areas of China as the region has vast coastline, abundant species, and suitable temperature. Bats are the only flying mammals which venture out every day and some of the species also retain seasonal migratory habits. Additionally, they as a viral reservoirs, by carrying viruses to different regions, including human habitats. A total of six sampling points along the southeast coast were selected, and the sequencing results were obtained for 2.37% tags ratio of assembled viral sequences. However, 95.73% of sequences were related to vertebrate virus which is far higher than previously reported percentages (8.97%-45%)^13, 14, 17^. This may be due to two reasons. First, reason includes the use of intestine and lung tissue from bats as samples for the virus metagenomics analysis; Before the advent of virus metagenomics, detection samples mostly consisted of bat faeces and throat swabs. This caused a large virus interference from the environment, and higher plant and insect virus ratio compared to this study^13, 17, 29^. In addition, due to the updated virus database, we had abundant reference sequences compared to previous studies. Through a complex comparison process, we annotated a total of 25 viruses, including 16 infected vertebrate viruses. This ratio is higher than previous research, but compared to the other studies fewer phage sequences were obtained. However, this result is limited by less sampling and may not reflect the real situation.

Six viruses closely related to human pathogenic virus species were selected for PCR verification of all 235 bat specimens. We found that the virus carrying rates and PCR results were consistent with the metagenomic sequencing results. For example, coronavirus sequence was only detected in ZS, CL, and SS group in the metagenomic sequencing results, while 11 positive amplications of coronavirus were obtained in PCR, including six cases in ZS group, four cases in CL group, and two cases in the SS group. During PCR detection of the circovirus, positive amplifications were detected in the ZS group, CL group, and LJ group, while metagenomic sequencing results only in the CL group notes to one tags. These differences may arise when metagenomic samples and PCR samples are amplified in different parts of the intestinal tissue or in bats with low viral load. Overall, the metagenomic sequencing results can provide strong technical support to classify virus spectrum. At the same time, these results also show that different bats carry different viruses in different regions. *Rhinolophus pusillus* can carry the most variety of viruses, including bocavirus, coronavirus, adenovirus, iflaviruse, porcine circovirus, astrovirus, and norovirus. Bats can carry a variety of viruses at a given time and different kinds of bats could carry different specific viruses. For example, in a collection of 55 Rhinolophus in the CL, norovirus, coronavirus, circovirus, and adenovirus were tested positive in *Rhinolophus pusillus*, while in *Rhinolophus ferrumequinum* only coronavirus tested positive. For future studies,it would also be interesting to study how different viruses exist at the same time without causing a pathogenic situation in bats.

After *Rhinolopus sinicus* was suggested to be the natural reservoir of SARS *coronavirus* which caused the 2003 pandemic, it was further confirmed that SARS-like virus strain SHC014-CoV isolated in China has the ability to re-emerge from viruses circulating in bat populations using the SARS-CoV reverse genetics system^30^. Coronavirus sequence was detected in *Rhinolophus pusillus* and *Rhinolophus ferrumequinum* in our study, but not in other bat species in ZS, CL, SS groups. PCR and metagenomic sequencing results were consistent. Coronavirus is the main host for *Rhinolophus* bat in China which is also consistent with previous research. Coronavirus has a high positive rate (6.8%-18.2%), especially in ZS and CL groups due to the amplification of limited fragments. However, for future research we will focus on the positive samples which detect full-length sequence amplification of coronavirus in bats.

Various types of adenoviruses of the genus *Mastdenovirus* infect a range of different mammals and cause respiratory, ocular, and gastrointestinal diseases^31^. The acquired partial sequence of the adenovirus CLR1/6/10 hexon from the three bats has the closest relation to bat mastadenovirus WIV9 (only 78.7% at the nucleic acid level) and porcine adenovirus 5 (only 63.1% nucleotide sequence identities); Moreover, our findings showed very close genetic relationships among the three bat adenoviruses CLR1/6/10 in *Rhinolophus pusillus*; although, they were divergent from the current mammalian adenoviruses. This indicates that the three bat adenoviruses CLR1/6/10 are new mammalian adenoviruses. Previous studies have reported both high prevalence rate and genetic diversity of astroviruses (AstVs) in Chinese bats^23, 32, 33^. All the bat AstVs in this study were clustered in a large clade together with previously reported bat astroviruses^23, 33, 34^, and a large branch of bat AstVs were distinct from other branches of human or ovine AstVs. The astroviruses detected in this study exhibited various degrees of sequence similarity with those described in previous studies. For example, bat astrovirus BatAstv/DSM1 showed 87.1% nucleotide sequence similarity with FJ571098 also found previously in Guangxi^34^, while bat Astv/CLR2, which we detected in *Rhinolophus pusillus* from CL, showed the nucleotide sequence similarity of only 66.6% with partial RdRp gene in known bat astroviruses, which suggested a novel species of bat astrovirus. Our findings suggest that astroviruses were genetically diverse not only within a single bat species but even within some individuals.

Among the known members of the family *Circoviridae*, PCV-2 is the only pathogen related to mammalian disease^35^. Circoviruses are commonly found in bats and show a large genetic diversity. Notably, Bt CV ZSR42 in our study shared the highest sequence identity with PCV, Bt CV CLR6, and Bt CV LJR22 clustered into avian Circovirus. This indicates that PCV has emerged, potentially as the result of a cross-species jump from birds into swine, most likely through intermediate contact with wild boars^36^. Bat bocaviruses were first identified in *M. myotis* in 2012 by Wu^13^. We first detected bat bocaviruses in the intestine of *Myotis davidii* and it was less than 85% homologous to known bat bocaviruses. Although large double stranded DNA viruses possess mutation rates far lower than RNA viruses, some small single-stranded DNA (ssDNA) viruses appear to mutate and have substitution rates closer to RNA viruses than double-stranded DNA viruses. These reports indicate that bocaviruses have potential cross-species transmission.

This study for the first time screened bats carrying norovirus in the coastal areas of China based on the metagenomic sequencing results. Norovirus sequences were detected in intestinal samples of *Rhinolophus pusillus* in ZS and CL groups and norovirus carrying rate reached 20% in ZS group. ZS has food rich in insects for bats. It is also easy for norovirus carrying bats to breed through faeces or direct contact with contaminated water or food. Although, there is no direct evidence that bats with norovirus can infect humans, norovirus carried by bat and the infection of human type GIV norovirus homology reached 76.8%, suggesting that bats may serve as norovirus natural host, a potential threat to human existence.

In conclusion, through the metagenomic sequencing of bat samples in southeast China, many mammalian viruses were annotated including some yet-unreported viruses. Furthermore, the PCR primers were designed according to the results of sequencing and the positive fragments were screened by PCR for phylogenetic analysis. We found that these viruses had low similarity with known viruses and suggest they could be categorized as new viruses. Also adenoviruses and circoviruses were also detected in *Myotis formosus* and *Myotis davidii* for the first time. However, further experiments are required to test if these viruses could infect humans. In addition, it is necessary to further investigate more samples in different locations to increase our understanding of the global diversity of bat viruses.

## Materials and Methods

### Ethics statement

The procedures for sampling of bats were reviewed and approved by the Administrative Committee on Animal Welfare of the Institute of Zhejiang and Fujian CDC Veterinary (Laboratory Animal Care and Use Committee Authorization). All live bats were maintained and handled according to the Principles and Guidelines for Laboratory Animal Medicine (2006), Ministry of Science and Technology, China. All animal experiments were approved by the Ethic Committee of Reasearch Institute for Medicine, Nanjing Command. All methods were performed in accordance with the relevant guidelines and regulations (Approval number: 2015009).

### Sample colletion and Nucleic acid extraction

After completion of collection from each sample site, all the bats were immediately dissected. The viscera of the sample was sub-packed in 1.5 ml EP tubes, labeled and placed in liquid nitrogen tank, shipped back to the laboratory, and put in −80 °C for cryopreservation until further process.

The sample was divided into six groups based on their location. The intestinal and lung tissues of the six groups were obtained and divided into 12 pools. A tip of every tissue (about 0.1 g) was placed in the pool. 10 times of SM buffer was added and every pool was ground until it was homogenized (50 mM Tris, 10 mM MgSO4, 0.1 M NaCl, pH7.5). The homogenate was centrifuged at 12000 g for 10 min at 4 °C but only the supernatant was used. The supernatant of each group was passed through 0.22 μm Pellicon II filters (Millipore, Billerica, MA) to filter out the broken tissue, bacteria, and other impurities. The filtrate was collected for nucleic acid digestion with 150 μl of following digestive products: filtrate 130 μl filtrate, 20U DNaseI (TaKaRa, Dalian, China), 5 μl 10 × DNaseI Buffer, 1 μl 10 mg/ml RNaseA (TaKaRa). The system was digested for 1 h at 37 °C. After digesting, the RNA was extracted with Qiagen RNAase minikit. The viral nucleic acid is used as reverse transcription template after suspending with 35 μl RNase-free H_2_O.

### Reverse transcription and random PCR

The nucleic acid was subjected to reverse transcription to get the first cDNA line of the total RNA by using a previously reported method^10^. Briefly, the miscible mixture which included 33 µl nucleic acid and 2 μl of 50 μM hexameric random primers labeled with 12 groups (20-bp tag sequences), was reacted in a water bath at 75 °C for 5 minutes, immediately put in ice bath for 2 minutes, and then mixed with 3 μl 10 mM dNTP, 20U RNAsin (TaKaRa), 5 × RT buffer, M-MLVRTase 5U (TaKaRa). The mixture was put in 42 °C for 1 hour to finish reverse transcription and get the first cDNA line. This was followed by a hot bath to inactivate the reverse transcriptase. The RNA in the mixture was mixed with 1U RnaseH at 37 °C for 30 minutes. The first cDNA mixture was purified by ethanol precipitation method.

### Double Stranded cDNA synthesis and sequence-independent single primer amplification (SISPA)

To obtain double stranded cDNA (dscDNA), exo-Klenow fragment (TaKaRa) and random primers were added for 60 min at 37 °C and then inactivated at 75 °C for 10 min. To obtain more viral nucleic acid products, dscDNA was subjected to SISPA amplification using the TaKaRa Extaq amplification system, and 20-bp tag primers (without hexamers) were used as the amplification primers in the above reverse transcription. A 25 μl reaction system containing 1 μl dscDNA mixture, 1 μl 20 mM tag primers, 10 × PCR Buffer, 4 μl 2.5 mM dNTPs, ExTaq Polymerase (1U), and ddH_2_O was denatured at 94 °C for 3 min, 30 cycles (94 °C for 30 s, 54 °C for 30 s, 54 °C for 30 s) and 72 °C for 5 min. The amplified products were purified using QIAquick PCR Purification Kit and dissolved in 50 μL TE buffer.

### Second-generation sequencing

The 12 groups of double-stranded DNA products were mixed into a sample labeled with different tag sequences and Illumina sequencing in one lane was performed by the Beijing Genome Institute (BGI, Shenzhen, China). 2.5-5 ng DNA was sheared into ~170 bp fragments by Covaris, and subjected to gel electrophotometry to examine the quality of the fragmented DNA. The fragmented DNA was combined with End Repair Mix and incubated at 20 °C for 30 min. The end-repaired DNA was purified with QIAquick PCR Purification Kit (Qiagen), the A-Tailing Mix was then added, and it was incubated at 37 °C for 30 min. The purified Adenylate 3′Ends DNA, Adapter, and Ligation Mix were combined and the ligation reaction was incubated at 20 °C for 15 min. Adapter-ligated DNA was selected by running 2% agarose gel to recover the target fragments. The gel was purified with QIAquick Gel Extraction kit (QIAGEN). Several rounds of PCR amplification with PCR Primer Cocktail and PCR Master Mix were performed to enrich the Adapter-ligated DNA fragments. The PCR products were then selected by running another 2% agarose gel to recover the target fragments. The gel was purified with QIAquick Gel Extraction kit (QIAGEN). The final library was quantitated in two ways: the average molecule length was quantified using the Agilent 2100 bioanalyzer instrument (Agilent DNA 1000 Reagents) and the library was quantified by real-time quantitative PCR (QPCR) (TaqMan Probe). The quantified libraries were amplified on cBot to generate the cluster on the flowcell (TruSeq PE Cluster Kit V3-cBot-HS, Illumina). The amplified flowcell was sequenced on the HiSeq. 2000 System (TruSeq SBS KIT-HS V3, Illumina) to generate 2 × 100 bp paired-end reads.

### Nucleotide sequence and maximum-likelihood phylogenetic analysis

The pre-product was sequenced according to Illumina standard technological process. The raw data was processed based on the internal program, including deleting the adapter and the host sequence, removing duplicated reads and a certain number of reads with low quality value (having > 2 N bases), and getting clean data. The reference database was built with internal procedures to extract the bacterial, fungal, archaeal organisms, and viral sequences from the nucleotide database, which was blasted with the sequences filtered by Short Oligonucleotide Analysis Package (SOAPaligner, version 2)^37^. Based on the following results, the sequence of reads with high correlation degree was given species classification. Different reads and sequences were merged for their high similarity and homology. In all the blast results, optimal results were used as the gene annotation with the parameter of E value < 10e-5. Functional analysis of all the genes was performed by BLAST alignment against KEGG (Kyoto Encyclopedia of Genes and Genomes) and eggNOG (Evolutionary genealogy of genes: Non-supervised Orthologous Groups) database.

### PCR to verify the virus sequence

Degenerate-nested primers (Supplementary Table S2) of the six viruses were designed for PCR validation, primer for cornovirus and astrovirus were synthesized according to previous reported^38, 23^, while primer for norovirus, adenovirus, bocavirus, and circovirus designed based on the of metagenomic sequencing results obtained in this study using Primer 5 (Premier Biosoft International, Palo Alto,CA). Nucleic acids of each individual sample were extracted using the QIAGEN kit. The PCR reaction system consisted of 18 μl PCR reaction solution (TAKARA), 0.5 μl of the upstream primer (10 μmol/L), 0.5 μl of the downstream primer (10 μmol/L), and 1 μl of the DNA template. PCR conditions were the following: pre-denaturation at 94 °C for 3 min; 30 cycles at 94 °C for 30 s, 52 °C for 30 s and 72 °C for 1 min for 30 cycles of in-house reaction; extension at 72 °C for 10 min. Positive PCR products were sequenced in both directions by an ABI 3730 DNA Analyzer (Invitrogen, Beijing, China).

### Evolutionary analysis

The positive sequence obtained by PCR amplification was aligned by BLAST in GenBank. The homologous sequences were downloaded from GenBank. The results were compared by MEGA6^39^ after comparing the sequences by ClustalW. Phylogenetic reconstructions were performed using MEGA6 and the maximum likelihood method or the neighbor-joining method was performed with 1,000 bootstrap replicates.

### Nucleotide Sequence Accession Numbers

The data from Solexa sequencing have been deposited in the GenBank Sequence Reads Archive under accession numbers PRJNA379515. All genome sequences of selected viruses have been deposited in GenBank under accession numbers KY775066 to KY775104.

## Electronic supplementary material

Supplementary Dataset
