# Supplementary material for: Virome analysis for identification of novel mammalian viruses in bats from Southeast China
Source: Sci Rep. 2017 Sep 7;7:10917. doi: 10.1038/s41598-017-11384-w (PMC5589946; doi:10.1038/s41598-017-11384-w)
Supplement: Supplementary file 1 — Supplementary Dataset [file 41598_2017_11384_MOESM1_ESM.doc]

**Supplementary Table S1.** Overview of viral tags

| Vertebrate |  |  | ZS | | DS | | XM | | CL | | SS | | LJ | | Total |
| --- | --- | --- | --- | --- | --- | --- | --- | --- | --- | --- | --- | --- | --- | --- | --- |
|  |  |  | intestine | lung | intestine | lung | intestine | lung | intestine | lung | intestine | lung | intestine | lung |  |
|  | Herpesviridae | Varicellovirus | 2 | 0 |  |  |  |  |  |  |  |  |  |  | 2 |
|  |  | Muromegalovirus | 2 | 0 |  |  | 0 | 2 | 0 | 4 | 1 | 0 | 0 | 3 | 12 |
|  |  | Proboscivirus |  |  |  |  |  |  | 1 | 0 |  |  |  |  | 1 |
|  |  | Macavirus |  |  |  |  |  |  | 0 | 1 |  |  |  |  | 1 |
|  |  | Rhadinovirus | 1 | 0 |  |  |  |  |  |  |  |  |  |  | 1 |
|  | Poxviridae | Orthopoxvirus |  |  |  |  | 0 | 3 | 1 | 3 |  |  | 1 | 1 | 9 |
|  |  | Parapoxvirus |  |  |  |  | 1 | 0 |  |  |  |  |  |  | 1 |
|  |  | Molluscipoxvirus |  |  | 2 | 0 |  |  |  |  |  |  |  |  | 2 |
|  |  | Avipoxvirus | 0 | 1 | 0 | 1 | 0 | 1 |  |  |  |  |  |  | 3 |
|  | Adenoviridae | Mastadenovirus | 21 | 0 |  |  | 0 | 1 | 15 | 0 |  |  |  |  | 37 |
|  | Astroviridae | Mamastrovirus |  |  |  |  |  |  | 1 | 2 |  |  |  |  | 3 |
|  | Circoviridae | Circovirus |  |  |  |  |  |  | 1 | 0 |  |  |  |  | 1 |
|  | Caliciviridae | Norovirus | 24 | 0 |  |  |  |  | 2 | 0 |  |  |  |  | 26 |
|  |  | Sapovirus |  |  |  |  |  |  | 3 | 0 |  |  |  |  | 3 |
|  | Hepeviridae | Hepevirus |  |  |  |  | 0 | 1 | 0 | 3 |  |  |  |  | 4 |
|  | Papillomaviridae | Alphapapillomavirus |  |  |  |  | 1 | 0 |  |  |  |  |  |  | 1 |
|  | Retroviridae | Betaretrovirus | 26 | 0 | 2 | 3 | 9 | 3 | 10 | 8 | 7 | 1 | 5 | 8 | 84 |
|  |  | Gammaretrovirus | 8 | 3 | 11 | 8 | 41 | 45 | 11 | 20 | 7 | 1 | 8 | 6 | 169 |
|  |  | Alpharetrovirus | 0 | 1 | 0 | 2 | 2 | 3 | 2 | 7 | 1 | 15 | 2 | 6 | 41 |
|  | Flaviviridae | Pestivirus | 0 | 1 | 0 | 3 | 0 | 42 | 2 | 4 |  |  | 0 | 2 | 54 |
|  | Parvoviridae | Iteradensovirus |  |  |  |  |  |  | 10 | 1 | 1 | 0 |  |  | 12 |
|  |  | Ambidensovirus | 1 | 7 | 1 | 0 | 1 | 0 | 6 | 1 | 0 | 1 |  |  | 18 |
|  |  | Bocaparvovirus | 13 | 0 | 28 | 0 |  |  | 35 | 0 | 1 | 0 | 2 | 0 | 79 |
|  |  | Dependoparvovirus | 10782 | 144 | 7 | 2 | 6 | 4 | 1209 | 648 |  |  | 0 | 2 | 12894 |
|  |  | Protoparvovirus | 208 | 0 |  |  |  |  | 22 | 1 |  |  |  |  | 231 |
|  | Togaviridae | Alphavirus | 2 | 0 | 0 | 1 | 2 | 0 | 0 | 1 |  |  | 0 | 1 | 7 |
|  | Picornaviridae | Parechovirus | 1 | 0 |  |  |  |  |  |  | 0 | 2 |  |  | 3 |
|  |  | Enterovirus |  |  |  |  | 91 | 1 | 16 | 0 | 1 | 0 | 6 | 1 | 116 |
|  | Coronaviridae | Betacoronavirus | 337 | 1 |  |  |  |  |  |  | 18 | 0 |  |  | 356 |
|  |  | Alphacoronavirus |  |  |  |  |  |  | 44 | 0 | 2 | 0 |  |  | 46 |
|  | Asfarviridae | Asfivirus |  |  | 1 | 0 |  |  |  |  |  |  |  |  | 1 |
|  | Reoviridae | Rotavirus | 3 | 0 |  |  |  |  | 1 | 0 |  |  |  |  | 4 |
|  | Subtotal |  | 11431 | 158 | 52 | 20 | 154 | 106 | 1482 | 704 | 41 | 20 | 24 | 30 | 14222 |
| Insect virus | Iridoviridae | Iridovirus | 3 | 0 |  |  | 0 | 1 | 2 | 1 |  |  |  |  | 7 |
|  |  | Lymphocystivirus | 12 | 0 | 2 | 0 | 8 | 50 | 9 | 12 | 5 | 0 | 26 | 3 | 127 |
|  |  | Ranavirus | 0 | 1 |  |  |  |  | 0 | 2 | 0 | 1 | 1 | 0 | 5 |
|  | Baculoviridae | Gammabaculovirus |  |  | 1 | 0 |  |  |  |  |  |  |  |  | 1 |
|  | Rhabdoviridae | Perhabdovirus | 1 | 0 |  |  |  |  |  |  |  |  |  |  | 1 |
|  |  | Tupavirus |  |  |  |  |  |  | 5 | 0 |  |  |  |  | 5 |
|  | Polydnaviridae | Bracovirus | 1 | 0 | 1 | 0 | 2 | 11 | 1 | 10 | 0 | 3 | 1 | 3 | 33 |
|  |  | Ichnovirus |  |  |  |  |  |  | 0 | 1 |  |  |  |  | 1 |
|  | Iflaviridae | Iflavirus |  |  |  |  | 4 | 0 | 65 | 0 |  |  |  |  | 69 |
|  | Subtotal |  | 17 | 1 | 4 | 0 | 14 | 62 | 82 | 26 | 5 | 4 | 28 | 6 | 249 |
| Plant virus | Luteoviridae | Polerovirus |  |  |  |  |  |  | 9 | 0 |  |  |  |  | 9 |
|  | Totiviridae | None |  |  | 8 | 0 |  |  |  |  |  |  |  |  | 8 |
|  | Virgaviridae | Tobamovirus |  |  |  |  |  |  | 5 | 0 |  |  |  |  | 5 |
|  | Phycodnaviridae | Chlorovirus | 2 | 0 | 3 | 3 | 3 | 2 | 4 | 4 | 2 | 0 |  |  | 23 |
|  |  | Phaeovirus | 2 | 0 |  |  | 0 | 1 |  |  |  |  |  |  | 3 |
|  |  | Prasinovirus | 1 | 1 | 1 | 1 | 0 | 4 | 2 | 4 |  |  | 1 | 0 | 15 |
|  |  | Coccolithovirus |  |  | 1 | 0 | 4 | 1 | 1 | 1 |  |  | 0 | 1 | 9 |
|  |  | Prymnesiovirus | 1 | 1 |  |  | 0 | 1 | 1 | 1 |  |  |  |  | 5 |
|  | Subtotal |  | 6 | 2 | 13 | 4 | 7 | 9 | 22 | 10 | 2 | 0 | 1 | 1 | 77 |
| Unclassified |  |  | 85 | 35 | 15 | 5 | 40 | 25 | 198 | 23 | 17 | 0 | 21 | 0 | 464 |
|  | Total |  | 11539 | 196 | 84 | 29 | 215 | 202 | 1784 | 763 | 65 | 24 | 74 | 37 | 15012 |

**Supplementary Table S2.** The nested primers for PCR or RT-PCR screening of each virus

|  | 1st Forward primer (5'-3') | 1st Reverse primer (5'-3') | 2nd Forward primer (5'-3') | 2nd Reverse primer (5'-3') |
| --- | --- | --- | --- | --- |
| Coronavirus | ATGGGWTGGGAYTAYCCIAARTG | TGYTGIGARCAAAYTCRTG | GGITGGGAYTAYCCIAARTGYGA | CCRTCATCWGAIARWATCATCAT |
| Adenovirus | TGGTCKTWCATGCACCATCG | ACATGGCCAGCACSTACTTYG |  | TCCACAGCYTGRTTCCAC |
| Astroviruses | GARTTYGATTGGRCKCGKTAYGA | GARTTYGATTGGRCKAGGTAYGA | CGKTAYGATGGKACKATHCC | GGYTTKACCCACATNCCRAA |
| Circovirus | GNCCNCCNCCNCAYAARMGNTGGT | NGGYTGCATNGCNGTYTGCC |  | CAYTTRTTNCKNGGNGGYTTC |
| Bocavirus | GGNGCNAARCARGCNAARATG | ATNGTNCCNGGNGGRTGYTCC | GGNGCNAARCARGCNAARATG | RTCRTTRTTNACCCANCC |
| Norovirus | GTCACCAAGAGAACYACTTTC | TCATAYTCYTCATCTGAGAGG |  |  |
